# How the seed coat affects the mother’s oviposition preference and larval performance in the bean beetle (*Acanthoscelides obtectus*, Coleoptera: Chrysomelidae, Bruchinae) in leguminous species

**DOI:** 10.1186/s12862-021-01892-9

**Published:** 2021-09-08

**Authors:** Árpád Szentesi

**Affiliations:** 1 Department of Systematic Zoology and Ecology, Loránd Eötvös University, Pázmány Péter sétány 1/C, Budapest, 1117 Hungary; 2 Department of Zoology, Plant Protection Institute, Hungarian Academy of Sciences, Herman Ottó út 15, Budapest, 1022 Hungary

**Keywords:** Leguminosae, Testa, Legume tribes, Egg-laying, Larval development, Preference, Performance

## Abstract

**Background:**

The host specificity and host range of the dry bean beetle, *Acanthoscelides obtectus* (Coleoptera: Chrysomelidae, Bruchinae), a seed predator of beans, is poorly known. In addition, the female oviposition preference and larval performance relationship is complicated by the respective importance of seed coat and cotyledon, because, paradoxically, females lay eggs on the basis of stimuli of the seed coat alone, without directly being able to assess the quality of the cotyledon’s suitability for larval development. Conversely, the thickness of seed coat may prevent first instar larvae from entering the seeds, even if cotyledons are suitable for development.

**Methods:**

The seeds of 62 leguminous species and 75 cultivars and accessions occurring in Hungary were evaluated for preference-performance relations. The preference of female bean beetles for seeds was measured in no-choice egg-laying tests. The ability of first instar larvae to overcome the seed coat as a physical barrier was tested with intact seed coat, while pre-drilled seed coats allowed the larvae to assess the suitability of cotyledon for development. The number of emerging adults was recorded. The thickness of seed coats and the weight of seeds were measured. Nonparametric tests and logistic regression were used for the statistical analyses of data and effect sizes were also calculated.

**Results:**

Seeds of 18 leguminous species (35% of them *Lathyrus*) supported larval development to adults if the seed coat was pre-drilled; however, only nine leguminous species supported development to adults if the seed coat was intact. Seed coat thickness beyond a critical threshold of 0.1 mm strongly influenced survival of first instar larvae. There was no overall positive correlation between oviposition preference and larval performance, except for 16 so-called acceptable non-hosts (Kendall’s τ = 0.3088). *A. obtectus* females also showed an ovipositional hierarchy of legume species even in no-choice tests.

**Conclusions:**

The results suggest that whereas the use of some acceptable non-host species by the *A. obtectus* is possible in seed stores, the same is unlikely under outdoor conditions, where the recognition of a diverse set of seed pod-related compounds would be necessary to induce egg-laying.

**Supplementary Information:**

The online version contains supplementary material available at 10.1186/s12862-021-01892-9.

## Background

In a seminal paper, Janzen [1] discussed the importance of seed coats and cotyledons of tropical legume species in their interaction with a bruchid beetle, *Callosobruchus maculatus* (F.). Based on no-choice oviposition and larval development investigations in the laboratory, he found extreme host selectivity of the species, which he attributed to the diversity of chemical compounds present in the hosts, and to physical traits, such as thickness, hardness and smoothness of the seed coat. However, more than forty years after Janzen’s paper, the host specificity of bruchids is still insufficiently known.

Although only implicitly, Janzen [1] also became one of the pioneers of female preference-larval performance studies, asking whether insect mothers optimally select substrates for larval development, and whether oviposition on novel or non-hosts are attempts of host range expansions [2, 3]. The latter hypothesis is supported by the fact that the range of plant species promoting survival of larvae is often wider than the egg-laying preference of the mother [4, 5]. The preference-performance theory postulates that females select egg-laying substrates in order to maximize their progeny’s fitness [3, 6, and references therein]. The female’s selection behaviour in a choice situation, where several items are presented simultaneously, and the female’s encounters with substrates of different quality is a sequential process, usually fits to the hierarchy of the genetically-based template of hosts [7]. Therefore, a positive correlation between the mother’s preference and offspring performance (usually the yield of adult offspring from a particular substrate) is probable. A meta-analysis of such studies [8], however, concluded that the results were contradictory: positive and negative correlations were equally abundant. Moreover, among the 23 studies of oviposition preference, there were only four no-choice tests. Females often make oviposition “mistakes”, e.g., laying eggs on toxic or otherwise unsuitable substrates; however, it is possible that these merely reflect the neural limitations of insects [9]. The strength of the relationship seems dichotomous according to the degree of host specialization of herbivorous insects. Specialists show strong positive preference-performance relationships, whereas in generalists, it may not exist at all [8, 10]. Several studies did not find genetic correlations between adult preference and larval performance either [11, 12]. The negative relationships refer not only to the lack of adaptation due to metabolic trade-offs, but to the importance of hitherto unconsidered life history components and environmental factors [8, 13, 14].

The preference-performance relationship arises in a different version for seed predator bruchid species. The complicating factor is the seed coat (testa, seed integument) that may or may not provide information on the quality of cotyledon for the egg-laying females or first instar larvae, but at the same time can impose formidable physical and chemical barriers for the larvae to enter the seed. The information on which the female bases her decision to oviposit may not adequately refer to the suitability of cotyledon, and the larva, following an exhausting period of drilling through a tough testa, may still die soon after contacting it. Despite the enormous advances in chemical analysis and the wealth of information of seed chemical composition in Leguminosae [15], relatively little is known of how seed coat chemistry affects host selection by bruchids.

Preference-performance studies evaluating a wide range of hosts and non-hosts are lacking due to the usually narrow host specialization of bruchids [1, 16]. Nevertheless, the bruchid species, *Acanthoscelides obtectus* (Say), *Callosobruchus* spp. and *Zabrotes subfasciatus* (Boheman) (Coleoptera, Chrysomelidae, Bruchinae), that are capable of propagating in storage on leguminous seeds, provide an opportunity to study host specificity. On a limited number of hosts, negative genetic correlations were found between preference and performance components in *C. maculatus* [17]. Furthermore, *A. obtectus* larvae reared on chickpea (*Cicer arietinum* L.) for ≥ 50 generations had lower survival rates in comparison to those reared on the natural host, *Phaseolus vulgaris* L. [18, 19]. It was concluded that there was a fitness cost for using an unusual host, and that whereas no genetic correlation was detected between preference and performance on *Phaseolus*, it existed on *C*. *arietinum*.

This paper investigates the potential host range and tests the preference-performance relations of *A*. *obtectus*. This species propagates on two species of cultivated beans (*P. vulgaris* and *P. coccineus* L.) in the temperate zone. In Europe, it can continuously reproduce on stored dry beans and therefore can achieve a pest status on this commodity. It also seasonally infests beans grown outdoors, laying eggs on nearly mature seeds within pods [20]. At harvest, infested seeds are collected, and the cycle is repeated in bean stores. Females do not stick eggs to the seed surface but place them among the seeds. First instar larvae have legs and can move among seeds to select sites on seeds to enter, and they can exercise independent selection behaviour, if a choice of seeds is available. While drilling through the seed coat, they avoid ingesting most of it [21], producing fine powder that indicates the site of boring. This behaviour is also observed with other bruchid species [1, 22, 23] and suggests the presence of potential chemical factors in the seed coat. Larvae tunnel into the cotyledon, develop through four instars and pupate within the seed.

The tribe Phaseoleae (Leguminosae) comprises ca. 89 genera, including *Glycine*, *Lablab*, *Vigna* and *Phaseolus* [24]. *A. obtectus* is an oligophagous species primarily attacking beans within the *Phaseolus* genus. However, spontaneous occurrence in some cultivars of the above three genera are also known, but it is a rare event and poorly documented, along with occasional observations concerning infestations in other legume genera such as *Lupinus* (tribe Genisteae), *Cicer* (Cicereae), *Vicia*, *Lens* and *Lathyrus* (Fabeae) [5, 25–28]. In seed storage facilities, only dry seeds are available, so selection of seeds for oviposition is dependent on the number of species being stored. Therefore, while selection choices for females can be limited, her oviposition decisions may be important for offspring survival.

With respect to *A. obtectus* there are several areas of research that have yet to be addressed, namely, to confirm host range, oviposition decisions under a no-choice environment and subsequent larval performance and survival. Females rank seeds by size [29], but are they able to judge their suitability for larval development based on information provided solely by the seed coat? The following hypotheses were also tested: (1) seed coat thickness determines penetration by first instar larvae, (2) ingestion of seed coat is toxic to the larvae, and (3) in the absence of the seed coat, the suitability of the cotyledon determines survival of larvae.

## Materials and methods

### The experimental insect

The test beetles originated from a continuous laboratory rearing maintained on commercial *P*. *vulgaris* beans for at least five years at 26–28 °C and 18/6-light/dark regime as described by Szentesi [30]. Following emergence, several hundred adults were placed into 10 × 15 cm size jars on corrugated paper, fed honey-water and allowed to mate. Adults are sexually mature at emergence from beans, and females can lay eggs by the third day. One-to-three-day old males and females were separated based on the shape and coloration of the pygidium and used for the experiments. To obtain eggs for the experiments, several hundred beetles were placed into an 18 cm high and 20 cm diameter glass cylinder, with the bottom opening capped with 1 × 1 mm mesh metal screen. The weevils laid eggs on a single layer of dry beans placed above the screen, and the eggs fell into a lower collecting dish when the cylinder was shaken (see more details in Szentesi [30]). As new beetles were put into the device daily, a continuous supply of eggs was provided. Daily collection of eggs allowed precise timing of emergence of the “black-headed” stage of first instar larvae. At this stage of development, the eggshell becomes transparent, and the head of the larva turns black in the egg, indicating the commencement of hatching of larvae within several hours, i.e., within 24 h at 26–28 °C.

### Plant species used in the tests

In this study, 62 species from the Leguminosae family belonging to the tribes Cercideae, Caesalpinieae, Genisteae, Amorpheae, Phaseoleae, Robinieae, Galegeae, Cicereae, Trifolieae and Fabeae, and 75 plant selections of six legume and four non-legume species (the latter as an outgroup) were tested for egg-laying and larval development (see Additional file 1: Tables S1, S2 and S3 for names of species, cultivars and accessions). These species either occur naturally in Hungary, are naturalized, or are cultivated as food or fodder. Only species with seeds which had masses large enough to support development of an *A. obtectus* larva were selected [31]. Seed mass was determined at room temperature with the help of an electronic balance (Sartorius A210P, Germany) operating with 0.1 mg accuracy.

As to developmental suitability of the plant species de Boer and Hanson’s [32] classification was used. *Hosts* (H) are those plant species that fully support development through generations and regularly harbour natural infestations. There are only two such species, the common bean (*P. vulgaris*) and the scarlet runner bean (*P. coccineus*) that fulfil these conditions in Hungary. *Acceptable non-hosts* (ANH) are not recognised and used as suitable substrates for larval development in nature. They grow usually asynchronously with bruchid phenology in space and time, and thus bruchid females are only occasionally constrained to use them, e.g., in seed stores. Although some adults may emerge, developmental time is typically long and larval mortality high. Chickpea is an example that belongs to this group. The third group, *non-hosts* (NH), comprises of plants that are nutritionally inadequate or toxic, and never supports development, although occasional egg-laying cannot be excluded in seed stores. The seed coat, the primary barrier to the cotyledon, was defined as *penetrable* if a first instar larva of *A. obtectus* was able to bore through it in case of an *intact* seed, and the cotyledon was considered *suitable* for development if an adult emerged from a seed regardless of whether the seed coat was intact or pre-drilled.

The seed samples originated from field or cultivated collections, and some were obtained from plant breeding companies. The samples were stored at a dry, cool place until use. Plant identification was carried out by the author.

### Egg-laying tests

Because no-choice egg-laying tests were carried out, “preference” is not used in the sense of choice tests, where selection by an insect is based on free movement among items offered, but instead refers to a position in the hierarchy of host-range. The measure of such preference for a plant species was the number of eggs laid/female on seeds. For the no-choice tests, the seeds of plant species were kept at 26–28 °C and 70–80% RH to allow them to take up moisture a week before the bioassay commenced. Thereafter, three seeds of the same plant line were placed into a 2 cm × 5 cm glass vial, and three 1–2-day old female and male adults introduced into each vial. A piece of white linen was fixed by a rubber band onto the vial to prevent adults from escaping. The vials were placed in a controlled environment chamber in darkness (L:D/0:24 h), and maintained until all weevils had died, and then number of eggs laid over the entire lifetime was counted. There were seven replicates for each plant species and selections.

### Larval performance test

Success of larval development on the different hosts was assessed by the yield of adult offspring. Seeds of all legume species and plant selections were handled as described in the previous section.

Two parallel treatments, with 45 replicates each, were set up. In one treatment the seeds were left intact, in the other they were pre-drilled with a high-speed electric drill (Triplex Miniplex, France) bit 0.14 mm in diameter, corresponding to the diameter of the first instar *A. obtectus* larva. One or two holes per seed were made at the ends and in the middle, respectively (depending on the size of seed) under a binocular microscope. Efforts were taken to drill through the seed testa only. Seeds were placed individually into glass vials (1 × 6 or 2 × 8 cm diam. × length) corresponding to seed size, and a single “black-headed” *A. obtectus* egg was placed onto the inner wall of the vial, ca. 1–2 cm above the seed, with the help of a fine wet brush, then the vial was capped with a cotton stopper. The vials were placed at 26–28 °C and 60–80% RH in dark conditions. To calculate the duration of time needed for development, the time of egg hatching and the L1s’ boring into the seeds were recorded by daily inspection from the beginning of the experiment. The observation of adult emergence was started after ca. 30 days. At least four months were allowed for development, then the seeds were opened under a dissecting microscope and the dead life stages recorded.

After establishing egg mortality, the remaining number of larval instars, such as L1 dead *outside* beans, plus dead L1, L2, etc. and pupae *inside* seeds, were taken as 100%; this value was then divided up among instars recorded. The instar of each larva was verified by head capsule measurement. All developmental malformations were also recorded. During dissection of the seeds, seed coat thickness was measured on ≥ 10 randomly selected seeds, using a micrometre under a microscope.

### Effect of bean seed coat on larval development

To test the hypothesis that the bean seed coat is not only a physical barrier against entering the cotyledon but also contains chemicals detrimental to the L1s, artificial beans (balls) were prepared. The ca. 150 mg size balls were prepared from finely pulverized cotyledons (seed coat removed) of *P. vulgaris* cv. Valja, and seed coat powder of the same cultivar was added in 2.5, 5 and 10% w/w concentrations. The seed mass of this bean variety is 240.7 ± 5.4 mg (mean ± SE, N = 35), and the seed coat is < 10% of the mass. The cotyledon was milled by a water-cooled grinder (Tekmar A-10, IKA, Germany) to avoid chemical deterioration from heat. The balls consisted of a standard amount of bean cotyledon powder (80%) mixed with 20% water-soluble potato starch. These balls served as controls. Adequate portions of the potato starch were substituted with seed coat to create the above concentrations, e.g., in case of balls containing 10% seed coat powder, there were 80% cotyledon and 10% starch powders. Addition of distilled water created a pastry from which 6 mm diam. balls were made by hand, and they were dried at 40 °C for a day. The acceptability of the artificial beans for egg-laying was tested with three males and three females and three balls for each treatment. To assess the balls’ suitability for larval development, one egg with a “black-headed” larva was placed on individual balls in vials in 13 respective replicates/treatment. The number of eggs laid, the percentage dead larval stages without or after boring into the balls, as well as that of emerging adults were recorded.

### Statistical procedures

Variance homogeneity of variables (larval mortality outside and inside seeds, and adult emergence from intact or pre-drilled seeds, etc.), notwithstanding various transformations, did not meet conditions of parametric tests, therefore nonparametric tests were used. Between-group comparisons were performed for three variables (e.g., plant groups) with Kruskal–Wallis ANOVA, and for two variables (e.g., intact and pre-drilled seed coat) with Wald-Wolfowitz runs test. The results are presented as medians and quartiles if data were obtained in percentages.

As a nonparametric version of discriminant function analysis, logistic regressions of the generalized linear models [33] were applied to reject or accept two major null hypotheses: (a) seed coat thickness does not influence L1 mortality, and (b) the suitability of seed cotyledon does not affect larval development. The continuous dependent variables indicated dichotomous distributions, therefore they were transformed to binary categorical variables at biologically reasonable cutpoints, and the coding of variables was performed accordingly: independent variables (*x*_*i*_), such as seed coat thickness, was assigned 1 if < 0.1 mm, and 0 if > 0.1 mm; cotyledon supporting larval development and adult emergence was given 1 if ‘suitable’, and 0 if ‘unsuitable’. Dependent variables (*y*_*i*_), such as L1 mortality outside seeds (N = 140), successful penetration of the seed coat (N = 140), larval development inside seeds (N = 212), and adult emergence (N = 212) were coded 1 if L1 mortality was < 50%, if L1 entered the seeds, if larval mortality inside seeds was < 30%, and if adult emergence from seeds was > 10%. Zero codes were assigned to the opposite outcomes (Additional file 1: Tables S6 and S7). Such zero–one “reference cell” coding [34] produced χ^2^-tables, where the left upper cells (A) were 11, and the right lower ones (D) were 00.

For logistic regression, STATISTICA ver. 6’s [35] GLZ module with binomial distribution, logit link function and sigma-restricted parameter estimation was used. The respective analyses used only a single predictor variable and one response variable. The program provided the estimate of the categorical predictor that maximized the probability of the dependent variable. For goodness of fit, Wald- and χ^2^-statistics of log-likelihood were calculated.

With the dichotomous data, effect sizes (risk difference, risk ratio, odds ratio and confidence intervals, CI) for L1 mortality and adult emergence were estimated by the methods described in [36, 37]. The calculations were based on 2 × 2 χ^2^-tables (Additional file 1: Tables S6 and S7). The odds ratio (OR) of the outcome was computed by the probabilities of cells: [*p*(1)/(1-*p*(1)]/[*p*(0)/(1-*p*(0)], where in *p*(1) the binary independent predictor is 1, and in *p*(0) it is 0. In order to be able to calculate effect size for adult emergence, 0.5 was added to all values in cells, because one element (adult emergence from NH plants) of the frequency table was zero. Interactions between dichotomous variables (e.g., ‘penetrable’/’impenetrable’ and intact/pre-drilled seed coat) were computed by multiplying two odds ratios (OR_10_ × OR_01_), assuming *H*_*0*_ (that there is no interaction) if the odds ratio cell OR_11_ equalled the result [38]. One-way ANOVA was used to evaluate the effects of concentrations of seed coat powder on larval mortality. All statistical procedures were performed with STATISTICA 6 [35].

## Results

### Female oviposition preference

**Fig. 1:**
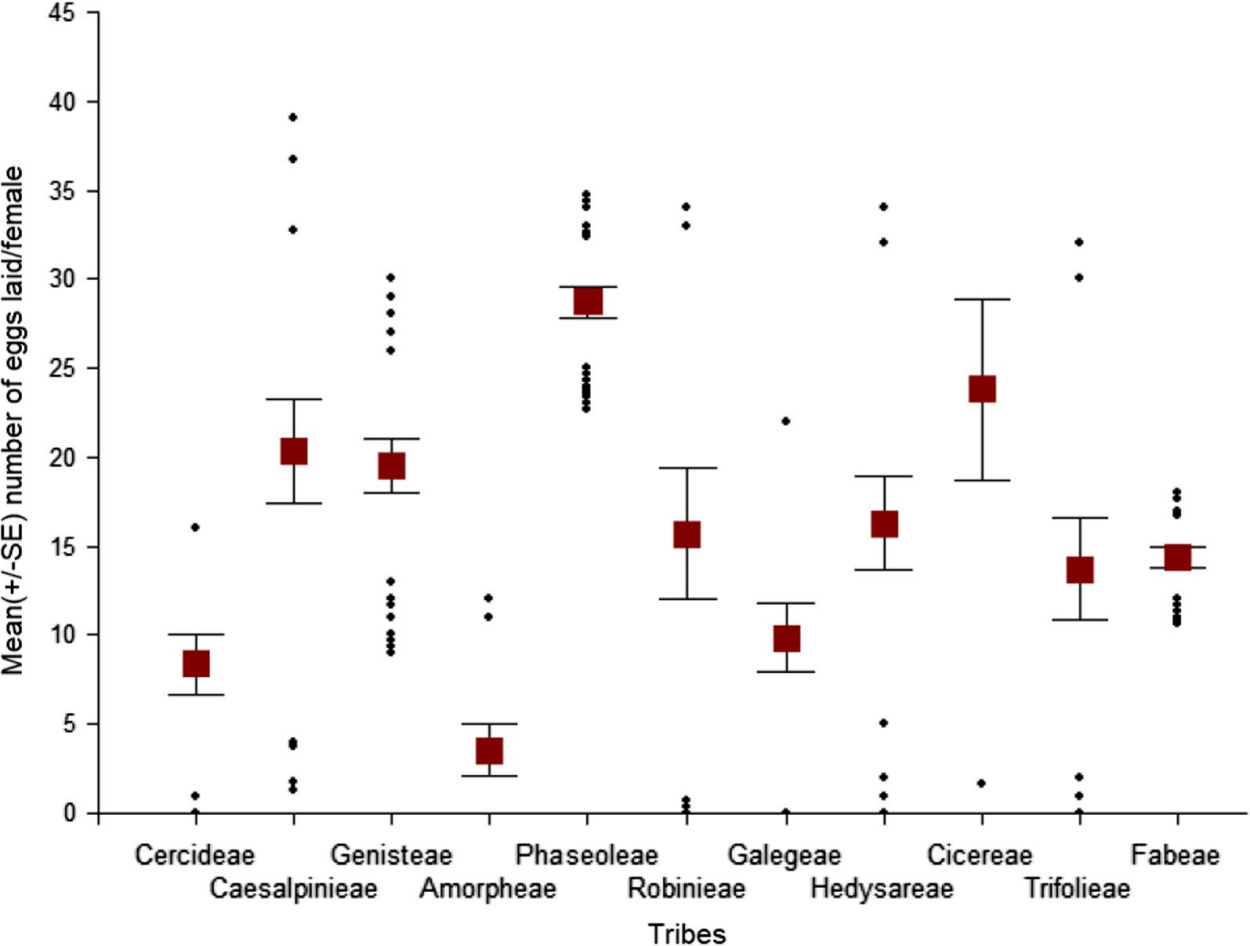
Distribution of the number of eggs laid/female *A*. *obtectus* across leguminous tribes. Data are means (± SE), points represent outlier values. The order of tribes corresponds to the phylogenetic relations presented by [24]

Bruchid females deposited significantly decreasing number of eggs/female on seeds in the order of host (H), acceptable non-host (ANH) and non-host (NH) species groups. Females laid a mean (± SE) of 38.6 ± 0.8 eggs on 22 H species and plant selections, 18.6 ± 0.7 eggs on 55 ANH species and plant selections, and 13.5 ± 0.5 eggs on 63 NH species and plant selections, and significantly more eggs on larger seeds (Table 1). Mean mass of H seeds was 340.8 mg, that of ANH 235.6 mg, and that of NH 70.3 mg (Table 1). All 66 plant species (62 leguminous and 4 outgroup species) received eggs, but females laid less than 15 eggs on 55%, less than 30 eggs on 35%, and between 30–45 eggs on 10% of plant species. Figure 1 shows the distribution of eggs among the leguminous tribes. Not surprisingly, *A. obtectus* females laid the highest number of eggs on members of the tribe Phaseoleae, where the main hosts are also found. Within Phaseoleae, soybeans (*Glycine*) were the least preferred (Additional file 1: Table S1). Comparable responses were noted to some species within the Caesalpinieae, Genisteae, Robinieae, Cicereae and Fabeae (Additional file 1: Table S1). Relatively high numbers of eggs were laid on some non-host species: *Gleditsia delavayi* Franch. (Caesalpinieae), *Laburnum alpinum* (Mill.) Bercht. & J. Presl, *L. anagyroides* Medik. (Genisteae), and *Robinia viscosa* Vent. (Robinieae). *C. arietinum* (Cicereae) and *Vicia faba* L. (Fabeae) are known as occasional hosts. The number of eggs laid/female on them fell into the medium and high categories, respectively. A mean number of less than 10 eggs/female were laid on *Vicia tenuifolia* Roth, *Robinia pseudoacacia* L. and *Amorpha fruticosa* L., and on some other members of the Fabeae tribe (Additional file 1: Table S1).

**Table 1:** Plant traits and responses of *A*. *obtectus* to seeds of host, acceptable non-host, and non-host leguminous species

Insect responses and plant traits	Hosts (H)		Acceptable non-hosts (ANH)		Non-hosts (NH)	
	Seed coat					
	Intact	Pre-drilled	Intact	Pre-drilled	Intact	Pre-drilled
L1-to-pupal mortality inside seeds (%)^1^	0a (0–2.2) (N = 22)	2.2A (0–2.3) (N = 22)	2.3b (0–7.7) (N = 55)	48.0B (24.4–82.8) (N = 55)	0c (0–0) (N = 63)	75.0C (44.0–93.0) (N = 63)
Adult emergence (%)^2^	75.3a (46.7–90.5) (N = 22)	93.3A (86.1–97.7) (N = 22)	0b (0–5.0) (N = 55)	26.7B (8.9–48.8) (N = 55)	0c (N = 63)	0C (N = 63)
L1 mortality outside seeds (%)^3^	24.7c (9.1–53.3) (N = 22)		93.3b (80.5–100) (N = 55)		100a (100–100) (N = 63)	
Number of eggs laid/female^4^	38.6 ± 0.8a (N = 154)		18.6 ± 0.7b (N = 418)		13.5 ± 0.5c (N = 630)	
Seed mass (mg)^5^	340.8 ± 4.3a (N = 773)		235.6 ± 3.9b (N = 1823)		70.3 ± 1.6c (N = 2052)	
Seed coat thickness (mm)^6^	0.09 ± 0.001c (N = 374)		0.10 ± 0.001b (N = 933)		0.13 ± 0.002a (N = 1098)	

Kruskal–Wallis ANOVAs: ^1^Intact seed coat: KW H’_2,140_ = 28.4, p < 0.001, pre-drilled seed coat: KW H’_2,140_ = 60.6, p < 0.001. ^2^Intact seed coat: H’_2,140_ = 93.6, p < 0.001, pre-drilled seed coat: H’_2,140_ = 120.9, p < 0.001; ^3^H’_2,140_ = 77.9, p < 0.001; ^4^H’_2,1202_ = 283.0, p < 0.001; ^5^H’_2,4648_ = 2515.2, p < 0.001; ^6^H’_2,2405_ = 216.2, p < 0.001. In comparisons between intact or pre-drilled seeds of plant groups, medians or means signed with different lower or upper case letters in the same row are significantly different, resp. ^1−3^Medians and quartiles; ^4−6^Means ± SEs. A comparison of adult emergence between intact and pre-drilled seeds of H plants was not significant (Z_adj_ = 0.7627, p = 0.4456), whereas comparisons between H_intact_ and ANH_intact_ (Z_adj_ = 6.1818, p < 0.001), between H_pre-drilled_ and ANH_pre-drilled_ (Z_adj_ = 5.6180, p < 0.001), and between ANH_intact_ and ANH_pre-drilled_ (Z_adj_ = 3.3525, p < 0.001) were significant (Wald-Wolfowitz Runs tests)

Females accepted the artificial seeds incorporated with seed coat as an oviposition substrate and laid similar number of eggs as on the control (Table 2).

**Table 2:** Egg-laying and larval survival of *A*. *obtectus* on artificial seeds incorporated with bean seed coat powder

Artificial seed treatment^1^	Number of eggs laid/female (mean ± SE)	Dead L1 outside (%)	Dead larvae inside (%)	Adults emerged (%)
2.5% seed coat	8.6 ± 2.1a	14.3	85.7	0
5.0% seed coat	4.5 ± 1.6a	28.9	71.1	0
10.0% seed coat	6.6 ± 1.2a	12.9	87.1	0
Control	7.6 ± 2.3a	2.7	2.7	94.6

^1^Artificial seeds (balls) consisted of 80% cotyledon powder plus 20% water-soluble potato starch powder. A portion of the latter was substituted with 2.5–10% seed coat powder. Control balls contained only cotyledon and starch powders. The Brown-Forsythe test indicated homogeneity of variances of the number of eggs laid/female: F_3,48_ = 1.5344, p = 0.2176, and one-way ANOVA (F_3,48_ = 0.8445, p = 0.4763), as-well-as the Scheffé post-hoc test were not significant at p < 0.05 (means signed with the same small letters)

### Larval performance

The major criteria of larval performance were: (a) L1 mortality outside seeds due to the thickness of seed coat, and (b) within-seed mortality of various developmental stages in the cotyledon. Both variables were modulated by the intact or pre-drilled status of seeds. The seed coat of host (H) and acceptable non-host (ANH) species was significantly thinner (Table 1). L1 mortality outside intact seeds was significantly lower for the H (24.75%) and ANH groups (93.3%), in comparison with the NH group (100%, all are medians, Table 1). For the two H and 16 ANH species, Table 3 provides data of seed coat thickness, whereas Additional file 1: Tables S2 and S3 give similar information for NH, and plant selections of H and ANH groups. Remarkably, seed coat constitutes a barrier even on the primary host, beans (*P. vulgaris*). Contrary to the very low larval mortality inside seeds, there was substantial mortality outside intact seeds of bean cultivars (27.3%, 13.6–53.3, median, lower and upper quartiles, N = 21), but only 4.4% (0–11.4) for pre-drilled seeds. Similar values can be given for other legume species from which adults emerged, with the difference that the upper level of mortality usually reached 100% with intact seeds, with some exceptions such as *Vigna unguiculata* (L.) Walp. and *V. angularis* (Willd.) Ohwi & H. Ohashi, where the seed coat was extremely thin (Tables 3 and 4). The best survival was seen in runner beans (*P. coccineus*), with 0% (intact seed) and 2.2% (pre-drilled seed) larval mortality outside the seeds, respectively.

**Table 3:** Mean (± SE) seed mass and seed coat thickness of leguminous plants, and the mean (± SE) number of *A*. *obtectus* adults emerged from species that supported development

Plant tribe and species^1^	Seed mass^2^ (mg) ± SE (N^4^)	Seed coat thickness^3^ (mm) ± SE (N^4^)	Number of adults emerged from 45 seeds (N^5^)	
			Intact	Pre-drilled
Genisteae				
* Lupius albus* L	197.2 ± 4.1 (46)	0.20 ± 0.007 (25)	0 (1)	3 (1)
Phaseoleae				
*Glycine max* (L.) Merr	167.9 ± 0.7 (910)	0.09 ± 0.001 (265)	0.5 ± 0.23 (17)	1.0 ± 0.45 (17)
*Lablab purpureus* (L.) Sweet	142.2 ± 3.5 (298)	0.15 ± 0.004 (24)	0 (1)	2 (1)
*Vigna unguiculata* (L.) Walp	109.6 ± 5.2 (70)	0.04 ± 0.002 (36)	28.5 ± 11.5 (2)	41.5 ± 1.5 (2)
*Vigna angularis* (Willd.) Ohwi & H. Ohashi	114.1 ± 1.7 (35)	0.07 ± 0.004 (20)	5 (1)	28 (1)
*Vigna radiata* (L.) R. Wilczek	69.1 ± 1.0 (35)	0.05 ± 0.001 (15)	0 (1)	39 (1)
*Phaseolus vulgaris* L	334.8 ± 4.4 (738)	0.09 ± 0.001 (359)	29.2 ± 2.3 (21)	39.7 ± 0.9 (21)
*Phaseolus coccineus* L	467.9 ± 8.3 (35)	0.09 ± 0.006 (15)	45 (1)	44 (1)
Cicereae				
*Cicer arietinum* L	189.6 ± 4.3 (35)	0.17 ± 0.004 (15)	1 (1)	44 (1)
Fabeae				
*Vicia faba* L	613.9 ± 10.1 (210)	0.18 ± 0.004 (110)	5 ± 2.2 (6)	17.5 ± 1.8 (6)
*Lens culinaris* Medik	48.7 ± 1.3 (105)	0.05 ± 0.001 (45)	0 (2)	1.0 ± 0 (2)
*Lathyrus hirsutus* L	26.8 ± 0.6 (35)	0.15 ± 0.003 (12)	0 (1)	5 (1)
*Lathyrus latifolius* L	57.3 ± 1.8 (35)	0.14 ± 0.006 (20)	0 (1)	1 (1)
*Lathyrus odoratus* L	54.0 ± 0.9 (33)	0.12 ± 0.006 (16)	0 (1)	3 (1)
*Lathyrus pratensis* L	15.0 ± 0.4 (35)	0.10 ± 0.002 (17)	0 (1)	7 (1)
*Lathyrus sativus* L	248.7 ± 7.0 (35)	0.09 ± 0.007 (15)	26 (1)	34 (1)
*Lathyrus tuberosus* L	37.0 ± 0.8 (70)	0.15 ± 0.003 (63)	0 (2)	10.0 ± 4.0 (2)
*Pisum sativum* L	243.2 ± 1.6 (975)	0.09 ± 0.001 (489)	1.7 ± 0.6 (27)	11.6 ± 2.0 (27)

^1^Species and authority names are given according to ILDIS (International Legume Database & Information Service) https://ildis.org/index.shtml [73]. Accessed Jan. 2021; ^2^Measured at room temperature; ^3^Measured under dissection microscope; ^4^Number of data evaluated; ^5^Number of species and cultivars/accessions

**Table 4:** Emergence and development time of *A*. *obtectus* adults and mortality of developmental stages in host and acceptable non-host seeds. Percentage data are presented as medians (see quartiles in Additional file 1: Table S4)

Plant tribe and species^1^	Adult emergence (%) from seeds		Development time (days)^2^		L1 mortality outside seeds (%)		L1-to-pupal mortality inside seeds (%)	
	Intact	Pre-drilled	Intact	Pre-drilled	Intact	Pre-drilled	Intact	Pre-drilled
Genisteae								
*Lupinus albus*	0	3.5			100	18.8	0	77.7
Phaseoleae								
*Glycine max*	0*	0*	68–101	67–92	97.7	2.3	2.2	95.5
*Lablab purpureus*	0	7.0			100	10.3	0	82.7
*Vigna unguiculata*	65.2	93.3	30–40	28–51	32.5	3.3	2.3	3.4
*V. angularis*	11.4	63.6	51–154	41–68	34.1	4.6	54.6	31.8
*V. radiata*	0	86.7		30–40	100	2.2	0	11.1
*Phaseolus vulgaris*	72.7	93.2	31–59	31–86	27.3	4.4	0	2.2
*P. coccineus*	100	97.8	37–44	37–44	0	2.22	0	0
Cicereae								
*Cicer arietinum*	2.2	97.8		33–58	97.8	2.2	0	0
Fabeae								
*Vicia faba*	6.7	38.4	32–68	35–79	83.9	35.6	4.7	19.2
*Lens culinaris*	0	2.3			100	51.2	0	48.8
*Lathyrus hirsutus*	0	20.0		84	100	32.0	0	48.0
*L. latifolius*	0	4.2		85	100	87.5	0	8.3
*L. odoratus*	0	12.0		85	100	32.0	0	56.0
*L. pratensis*	0	46.7		84	100	6.7	0	46.7
*L. sativus*	57.8	75.6	33–90	33–47	40.0	11.1	2.2	13.3
*L. tuberosus*	0	13.6		40–42	100	28.0	0	44.4
*Pisum sativum*	0	20.5	46–101	40–162	93.2	7.3	4.6	62.5

^1^According to ILDIS (International Legume Database & Information Service) https://ildis.org/index.shtml [73]. Accessed Jan. 2021; ^2^From L1’s entering the seeds to adult emergence (min–max values). Empty cells mean missing data. *Although the medians were zero, a low number of adults emerged from seeds (see Table 3)

Mortality of various developmental stages inside intact seeds were substantially different from that within pre-drilled ones; however, the critical event for development in the seeds was invariably the survival of first instar larvae. Tables 1 and 4 provide results for H and ANH species, and further data are available in Additional file 1: Tables S2 and S3 for bean, pea and soybean varieties, as well as for NH species, showing differences among host types. Because larvae were unable to penetrate the intact seed coat of NH species, there was no larval establishment inside NH seeds. In comparison, larval establishment was 75% (44–93%, median and quartiles) within pre-drilled seeds. It was observed with all plant groups that many larvae entered seeds via the pre-drilled hole but then exited and died. Seed testa frequently had several shallow pits, where L1 attempted to bore in (e.g., all *Gleditsia japonica* Miq. seeds, both intact and pre-drilled, had such traces). Although it is well documented that additional *A. obtectus* larvae may enter through the hole made by a pioneer larva [39], the ratio of L1-made and artificial holes was 1.5:1 on the most preferred bean seeds (*P. coccineus*), i.e., many L1 larvae did not use the pre-prepared holes on this host. In some instances (e.g., *Caragana* or *Onobrychis* genera), L1s entered seeds through the hilum. First instar larvae entering *Gleditsia* seeds through an artificial hole made at the embryo area invariably died within the embryo. In cases where the cotyledons were soft (several *Glycine* cultivars/accessions and *Caragana*), larvae made longer tunnels before dying.

The experiment that aimed to elucidate the effects of seed coat of beans on larval development proved that, besides being a physical barrier, the seed coat also inhibited larval development at the lowest concentration incorporated into artificial seeds (Table 2). Conversely, controls (without any seed coat content) were fully suitable for development.

Significantly more adults emerged from H species and plant selections than either from ANH species and plant selections or NH species, and whether intact or pre-drilled (Table 1). Of the 62 legume species, *A. obtectus* larvae developed into adults in 18 (29%) species in four tribes, if the seed coat was pre-drilled, compared to only nine species if they had intact seed coat (Table 4). However, the picture varied considerably concerning plant selections (Additional file 1: Table S3). Although adult emergence in *P. vulgaris* was generally high, at the cultivar level it ranged between 51 and 100%. In *P. coccineus*, all larvae developed into adults. Interestingly, even intact cowpea (*Vigna*) seeds supported larval development to adults, whereas in white lupin (*Lupinus albus*), adults emerged from pre-drilled seeds only. Pre-drilled soybean (*Glycine*) plant selections and hyacinth bean (*Lablab*) seeds assisted higher survival to adults than intact seeds. This is also paralleled by the length of developmental time needed until adult emergence (Table 4); for instance, it was two to three times longer in *G. max* (L.) Merr., *V. angularis* and *P. sativum* L. in comparison with beans. Of the 27 plant selections of garden peas, adults emerged from 24 (88.9%), however, only from 13 (48.1%) of these if the testa was intact. Similar values occurred for 17 *G. max* plant selections: adult emerged from six (35.3%) of them, but only from four of these with an intact seed coat. A surprising feature is the asymmetric distribution of adult emergence between the *Vicia* and *Lathyrus* genera.

Of the 18 leguminous species that had pre-drilled seed testa and were suitable for survival through to adults, in five (28%) species some adults that emerged showed malformation. Furthermore, in 10 leguminous species, larvae developed through to adults but were unable to emerge. Typical malformation was a substantial decrease in elytra width and length: the elytra became shorter and triangular. There were 0.1% malformed adults in beans, 33.0% in peas, 31.6% in *L. tuberosus* L., 6.7% in *L. sativus*, and 0.7% in *V. faba*.

### Female preference versus larval performance

Additional file 1: Table S5 provides the most important nonparametric correlation coefficients referring to the overall relationship between plant traits and insect responses. Accordingly, there was a significant positive relationship between preference and performance only in the ANH group, i.e., between the number of eggs laid/female and adult emergence (Kendall’s τ = 0.3088, intact seeds, N = 55). The correlations between seed mass and the number of eggs laid were extremely low in all three plant groups but were significant in ANH and NH species. The thicker the seed coat, the higher was the first instar larval mortality outside on ANH and NH seeds, and seed coat thickness also affected L1 larval mortality on H seeds too.

The logistic regression provided evidence that L1 mortality outside seeds was due to different seed coat thicknesses. With the condition of less than 50% L1 mortality outside seeds, 15.6% (7.1–32.6, median and quartiles, N = 19) mortality occurred if the seed coat was thin (0.08 ± 0.001 mm, mean ± SE, N = 78), in comparison with 100% (97.8–100, median and quartiles, N = 61), if the seed coat was thick (0.15 ± 0.005 mm, N = 62) and with mortality higher than 50%. (Wald test for seed coat thickness was 8.2, df = 1, p = 0.0043; log-likelihood: -48.4; goodness of fit χ^2^: 17.9, df = 1, p < 0.001.) Approaching the same hypothesis from another angle, i.e., if the seed coat was assigned as’penetrable’ or ‘impenetrable’, significantly higher number of L1 entered seeds with ‘penetrable’ seed coat (Wald test: 27.3, df = 1, p < 0.001; log-likelihood: -81.4; χ^2^ = 31.1, df = 1, p < 0.001). Here, only those cases were considered where L1 larvae entered a seed then died immediately after it. This result is interesting, because ‘penetrable’ seed coat thickness was 0.0998 ± 0.004 mm (N = 64), whereas ‘impenetrable’ was 0.119 ± 0.005 mm (N = 76), a mere 0.02 mm difference.

Larval performance and adult emergence did depend on the ‘quality’ or ‘suitability’ of seed cotyledons. With the criterion of less than 30% larval mortality inside seeds, mortality was 2.27% (0–7.33, median and quartiles, N = 87) if the cotyledon was ‘suitable’ for larval development, in comparison with ‘unsuitable’ cotyledons (80.0%, 60–95.5, median and quartiles, N = 60), and with mortality higher than 30%. (Wald test for cotyledon suitability was 27.6, df = 1, p < 0.001; log-likelihood = -131.5; χ^2^ = 29.8, df = 1, p < 0.001.) Adult emergence from ‘suitable’ seeds was 37.8% (10–84.1, median and quartiles, N = 123) *vs.* from’unsuitable’ cotyledons: 0% (N = 89), thus logistic regression for adult emergence could not be performed. Nevertheless, effect sizes could be computed from the χ^2^ table.

Effect sizes and risk analyses showed that L1 larvae had 19 times larger chance to have < 50% mortality outside seeds by odds ratio (OR). Risk difference (RD) indicated 45% higher survival for larvae if they happened to bore in a seed with seed coat thickness of < 0.1 mm, in comparison with seeds having thicker testa (Additional file 1: Table S6). However, the regression coefficient (*φ*^2^) explained only 10% of variance of seed coat effect, referring to other important factors affecting larval entry. On the other hand, when facing penetrable/impenetrable seed coat (results are not shown in table), L1 larvae had three times higher risk to die facing ‘impenetrable’ seed coat (RR = 3.01 ± 1.3, mean ± SE, CI_95_ = 1.9 & 4.8). However, chances for larvae to enter a seed with penetrable testa was 7.5 times higher (OR = 7.5 ± 1.5, CI_95_ = 3.5 & 16.1), even if they died after the first bites from the cotyledon. Here, the regression coefficient explained a relatively high level (21%) of the variance.

The risk of > 30% larval mortality inside seeds increased ca. 2 times (RR = 1.95 ± 1.13, CI_95_ = 1.52 & 2.49) in cotyledons unsuitable for reaching later developmental stages. The odds for such an outcome were high (OR = 4.85 ± 1.28, CI_95_ = 2.96 & 7.94, results are not shown in table). The chance for reaching adulthood in seeds from which > 10% adults emerged was ca. 4 times higher (RR in Additional file 1: Table S7), than in cotyledons allowing only 1–2 weevils to successfully complete their development. The odds for adulthood in suitable seeds were extremely high (OR = 523.42 ± 1.23, CI_95_ = 348.74 & 785.60) due to the asymmetry caused by the NH seeds. The regression coefficient (*φ*^2^) explained a high portion (54%) of the variance (Additional file 1: Table S7).

As expected, there was an interaction between penetrable/impenetrable and intact/pre-drilled states of seed coats: the joint effect (OR_11_ = 0.6718) was larger than the multiplied value (0.4820) of their respective effects (OR_10_ = 0.7179 and OR_01_ = 0.6714). The joint effect of both variables is 1.4 times higher than the combined effect of each variable acting separately.

## Discussion

This study could not prove an overall positive correspondence between host preference and host suitability. However, 16 leguminous plant species (Table 4) did show positive significant relationship between oviposition preference and larval performance (Additional file 1: Table S5), and these are called *acceptable non-hosts* (ANH) [32]. Nine of these legume species supported development into adult stage even if the seed coats were intact. Besides the two *Phaseolus* species, rearing in laboratories is possible on chickpea through generations [18, 40], and occasional infestations occur in stores on garden peas or faba beans (Szentesi unpubl. results). How females assess host suitability of ANH species through the seed coat remains an intriguing aspect of the positive relationship between female choice and larval success. The distribution of larval mortality and adult emergence (Tables 1 and 4, Additional file 1: Tables S2 and S3) among plant groups, and the results of logistic regression (Additional file 1: Table S6), overwhelmingly emphasize the importance of the seed coat in allowing or preventing the use of a seed. For instance, regression coefficients (*φ*^2^) explained 10% and 21% of the variance, respectively, of the effect of seed coat thickness and ‘penetrability’, supporting the hypothesis that several yet unknown physical and chemical factors are also important. In spite of the fact that a preference for larger H seeds has been proven in bruchids in choice-tests [29, 41], the seed mass of plant groups had negligible effects on the number of eggs laid/female (Additional file 1: Table S5), and the significant positive preference relationship in ANH and NH species might be attributable to physical traits, e.g., curvature of the seed surface.

In the present study only a limited number of possible variables that may affect preference-performance relationships (seed mass, number of eggs laid/female and seed coat thickness) was taken into consideration. Other important factors (e.g., chemicals, further physical, environmental, life history traits, mobility, predators, host genotype) that influence host selection [8, 10, 42] were not investigated. In addition, experience may promote acceptance of ANH species [43, Szentesi unpubl. results] and may even lead to host-race formation [44], but neurological constraints can impose limitations to perception and integration of stimuli [9]. In the light of many factors influencing preference-performance relationship, and specifically egg-laying, it is doubtful whether the oviposition response of *A. obtectus* females on ANH and NH species was “erroneous” or “defective”, as there are possible alternative explanations for the behaviour [45, 46].

### Is there a hierarchy in host selection in no-choice tests?

The widely known hierarchy-threshold model [7] for individual host selection in choice situations explains that specificity is bound to genetic and physiological status, and that the rank-order of hosts is invariable. Oviposition occurs when acceptability of a substrate exceeds the motivational threshold, and the different acceptability levels create a rank-order. An alternative hypothesis [47] states that it is the actual set of eggs available for laying that decides acceptance: higher number of eggs motivate acceptance of low-quality hosts. In this study, however, no-choice tests were applied. *A. obtectus* females did produce a rank-order of ANH plants; however, except for the primary host beans, this was different from those obtainable in choice tests, and even more different concerning larval development (Szentesi unpubl. results, Additional file 1: Table S8). In choice tests, *A. obtectus* visits items in a sequential way and lays most eggs on beans. However, it never totally ignores other substrates (even glass beads), because variation in egg-laying is also modulated by factors such as egg-load and/or, in most cases, unknown physical and chemical stimuli. In this study females laid ca. 1/3 of the number of eggs on NH species compared to those deposited on H plants. This means that elements of acceptability of different NH plants could induce the motivational state to accept a lowered egg-laying threshold as time passed.

### The host-range of *A. obtectus*

Pest status, width of geographic distribution and host range can be interconnected [48]. *A. obtectus* is narrowly specialized on some wild and cultivated members of the genus *Phaseolus* [49], but it is a widespread and important pest, whose host affiliation may also include some species of pantropical leguminous tribes [50]. In the present study, *P. vulgaris* and *P. coccineus* represented the hosts, the second providing better preference/performance ratio as also confirmed by [25]. Some species and cultivars of the following genera belong to the ANH group: *Lupinus*, *Glycine*, *Lablab*, *Vigna*, *Cicer*, *Vicia*, *Lens*, *Lathyrus*, and *Pisum* (Table 4), largely agreeing with findings of [5, 16, 26, 27]. Differences depend on whether the authors applied a sufficiently wide spectrum of plant selections, because differences in acceptance among these can be as large as, or larger than, between species (Additional file 1: Tables S2 and S3).

Pre-drilled seeds contributed to the understanding of host-range by demonstrating how important chemical constituents of the cotyledons were when seed coats did not function as barriers. The surprisingly high ratio of *Lathyrus* species supporting development to adulthood (Table 4) could be possible only in case of pre-drilled seed coats. It is noteworthy that most of those plant species that allowed some larval development were members of the tribe Fabeae, and specifically of the genus *Lathyrus* and much less of *Vicia* (Additional file 1: Table S2).

### The importance of seed coat

The legume seed coat is a significant barrier. In Janzen’s [1] work, no bruchid larva was able to penetrate through > 0.4 mm thick seed coat. From another perspective, Thiery [51, 52] demonstrated that seed coat hardness coupled with low (ca. 6%) moisture content caused higher than 90% L1 mortality with *A. obtectus* on *P. vulgaris*. The high L1 mortality as a result of intact seed testa in case of plants grown in nature in this study (Additional file 1: Table S2) calls attention to further factors. These complementary findings indicate that both physical (thickness, smoothness and hardness) and chemical traits of the seed coat contribute to mortality, and it seems that no study has attempted to separate the respective effects.

### Chemical factors in the seed coat

One obvious factor that should direct the attention to phytochemicals in seed coats is the observation with several bruchid species [1, 22, 23], *A. obtectus* included [21, Szentesi pers. obs.], that L1 larvae consume little or none of the seed coat. It was also demonstrated by Stamopoulos and Huignard [53], and in this study (Table 2), that the consumption of bean seed testa was toxic to *A. obtectus* larvae. A multitude of chemical factors have been identified in legume seeds coats [15]. For example, *A. obtectus* L1s did not even attempt to penetrate the intact seed coat in the Genisteae tribe, due probably to quinolizidine alkaloids that are present in the testa [54].

### Chemical factors in the cotyledon

The cotyledon ultimately determines whether larval development can reach the adult stage. A wide spectrum of secondary plant substances occurs in leguminous tribes, but one of the most characteristic groups is non-protein (also called toxic) amino acids [55]. Not only are their remarkable amounts inside seeds (up to 8% of dry weight [56]) important, but also is their taxonomic distribution in Fabeae. The cotyledon of most *Lathyrus* species contains a diverse array of toxic amino acids, most frequently homoarginine and lathyrine, whereas *Vicia* species can be characterized by the dominance of canavanine [57, 58]. In this study, within the Fabeae tribe, *V. faba* was the only species that allowed development of *A. obtectus* to adulthood (Table 4). Canavanine can be a significant factor influencing survival of *A. obtectus* in NH *Vicia* seeds, in spite of the fact that *A. obtectus* larvae show a remarkable tolerance to this compound: at 2.0% w/w concentration some adults (4% of the control) still emerged from artificial seeds (Szentesi unpubl. results). However, toxic amino acids occur in many other legume species and they act in combination with several other chemical groups to form the chemical resistance profile of a seed [59]. Indicators of the effects of this complex milieu are malformations and slower development, presumably consequences of the higher metabolic costs of handling substances in the cotyledon.

### Evolutionary considerations

The seed coat not only excludes potential exploiters; it also effectively divides the “perception space” of egg-laying insects. Whereas insects can directly perceive plant quality by probing in cases of leaves and fruits, *A. obtectus* females seem to be unable to judge suitability of cotyledons by the seed coat. The possibility exists that the seed coat can mediate information on the inner quality of the seed; however, the stimulus complex leading to egg-laying on an unsuitable seed is not known. The outcome of preference-performance conditions is further complicated in the field. Although *A. obtectus* was able to lay eggs into *V*. *unguiculata* pods in the laboratory [28], this would not necessarily happen in nature. These findings also direct attention to the need of more tests with pods of different ANH plant species, as this must be the first step to host-range expansion.

An intriguing result of this study was the surprisingly high frequency of development of *A. obtectus* to adulthood reared on meadow peas (*Lathyrus* species), when seed coats were pre-drilled. There are no reasons for assuming any evolutionary connection in the relationship, because (1) larvae cannot enter *Lathyrus* seeds with intact testa, (2) some *Lathyrus* species do not contain toxic substances to *A. obtectus* larvae by chance or domestication, and (3) *A. obtectus* females most likely do not recognise the suitability of *Lathyrus* seeds for larvae, as they assess only pods for egg-laying in nature. (During the 30 years of collecting legume seeds from *Lathyrus* species from the field, *A. obtectus* has never been reared, Szentesi and T. Jermy unpubl. obs.)

As in several other instances [60], the egg-laying by *A. obtectus* females onto ANH seeds also raises the question as to whether host range expansion has occurred. The first steps in host shifts are behavioural events [61–63], viz*.* the ultimate conditions are recognition and acceptance of the new plant species as host. Although oviposition preference and larval performance likely are governed by different gene complexes [4, 64, 65], there must be genetic covariance between preference and performance to adapt to a new plant species [66, 67]. Egg-laying should be accompanied by physiological adaptations of larvae to handle compounds metabolically once they are inside seeds [68, 69]. Unless preadaptation [70] played a role, as was recently suggested with a related species (*A. macrophthalmus*) [71, 72], host shift with *A. obtectus* is less likely. The behaviour of not ingesting the seed coat while boring in, observed with L1 larvae of several bruchid species, can be adaptive. It ensures that, in case of a host shift, larval acceptance or rejection behaviour would be related to the chemicals of cotyledon only.

The following traits maintain *A. obtectus* within its current status of host specialization: (a) first instar larvae are not constrained to enter an acceptable non-host seed in a no-choice environment, because they have legs, and as long as their energy reserves allow it, they can actively seek suitable hosts; (b) first instar larvae rarely enter acceptable non-host seeds with intact seed testa; and (c) the life cycle of *A. obtectus* takes place both in stores and in the field, and this spatial segregation regularly interrupts possible breeding and selection on potential additional hosts. Even if generations of beetles were produced on acceptable non-host seeds, the recognition of these plants as suitable oviposition sites must happen based on traits of pods (not seed coats) in the field that, as a first step, would require substantial genetic changes.

## Conclusions

This study provides evidence that the seed coat of leguminous plant species hampers both A. *obtectus* females from judging the suitability of the cotyledon for successful larval development and neonate larvae to enter seeds. Although females are prone to oviposit on dry seeds of many leguminous plant species, the L1 population suffers high mortality while drilling through the seed testa. Making the seed coat penetrable by artificial holes exposed the larvae to possible chemicals factors in the cotyledon that resulted in additional mortality with an exception of 16 leguminous species designated as’acceptable non-hosts’. Apart from this plant group, the female preference and larval performance relation was negative. Further investigations should clarify the role of chemicals of seed testa and cotyledon of leguminous plants in their acceptance and rejection by females and neonate larvae.

## Supplementary Information

**Additional file 1.** Additional tables.

## Data Availability

The datasets used and/or analysed during the current study are available from the author on reasonable request. Additional information is available in Additional file 1: Tables.

## Abbreviations

H: Host
ANH: Acceptable non-host
NH: Non-host
KW: Kruskal-Wallis
OR: Odds ratio
RD: Risk difference
RR: Risk ratio
CI: Confidence interval
GLZ: Generalized linear model
