# Supplementary material for: How the seed coat affects the mother’s oviposition preference and larval performance in the bean beetle (Acanthoscelides obtectus, Coleoptera: Chrysomelidae, Bruchinae) in leguminous species
Source: BMC Ecol Evol. 2021 Sep 8;21:171. doi: 10.1186/s12862-021-01892-9 (PMC8425149; doi:10.1186/s12862-021-01892-9)
Supplement: Supplementary file 1 — Additional file 1. Additional tables. [file 12862_2021_1892_MOESM1_ESM.pdf]

BMC Ecology and Evolution

How the seed coat affects the mother's oviposition preference and larval performance in the bean beetle (*Acanthoscelides obtectus*, Coleoptera: Chrysomelidae, Bruchinae) in leguminous species

Árpád Szentesi

Department of Systematic Zoology and Ecology, Loránd Eötvös University, Budapest, Pázmány Péter sétány 1/C, H-1117, and Department of Zoology, Plant Protection Institute, Hungarian Academy of Sciences, Budapest, Herman Ottó út 15., H-1022, Hungary; arpad.szentesi@ttk.elte.hu

Supplementary Tables

Additional file 1: Table S1 Mean (±SE) number of eggs laid/bean beetle (*Acanthoscelides obtectus*) female on seeds of leguminous and non-leguminous species in no-choice tests. Species-level also includes oviposition responses to plant selections (cultivars and accessions, where such were available), genus-level shows the mean number of eggs laid on all species, and tribe-level represents averages of genera

Plant Family, Tribe, Genus and Species	Egg/female			Species status
	at Species-level	at Genus-level	at Tribe-level	
CAESALPINIACEAE				
**Cercideae**				
Cercis canadensis L.[1]	7.9±2.2			NH
C. siliquastrum L.[2]	8.7±1.4	8.4±1.2	8.4±1.2	NH
**Caesalpinieae**				
Gleditsia delavayi Franch.[1]	32.6±2.2			NH
G. japonica Miq.[1]	13.9±3.3	20.3±2.1	20.3±2.1	NH
G. triacanthos L.[2]	14.6±2.7			NH
FABACEAE				
**Genisteae**				
Cytisophyllum sessilifolium (L.) O. Lang[2]	13.2±2.9	13.2±2.9		NH
Cytisus scoparius (L.) Link	16.5±3.7	16.5±3.7		NH
Laburnum alpinum (Mill.) Bercht. & J. Presl	34.9±3.6			NH
L. anagyroides Medik.	23.3±2.5	29.1±2.3		NH
Lupinus albus L.[3]	17.3±2.4		19.5±1.1	ANH
L. multiflorus Desr.[3]	19.6±3.1	18.8±1.8		NH
L. polyphyllus Lindl.[2]	20.8±4.0			NH
Petteria ramentacea (Sieber) C. Presl[2]	12.1±2.5	12.1±2.5		NH
Spartium junceum L.[2]	18.7±3.1	18.7±3.1		NH
**Amorpheae**				
Amorpha fruticosa L.[2]	3.5±1.0	3.5±1.0	3.5±1.0	NH
**Phaseoleae**				
Glycine max (L.) Merr.[4]	17.4±0.7	17.4±0.7		ANH
Lablab purpureus (L.) Sweet[4]	19.7±2.8	19.7±2.8		ANH
Phaseolus coccineus L.[5]	44.1±2.2			H
P. vulgaris L.[5]	38.4±0.8	38.6±0.8	26.9±0.6	H
Vigna angularis (Wild.) Ohwi & H. Ohashi[4]	25.3±2.0			ANH
V. radiata (L.) R. Wilczek	26.3±3.7	27.6±1.5		ANH
V. unguiculata (L.) Walp.[4]	29.3±2.1			ANH
**Robinieae**				
Robinia pseudoacacia L.[2]	2.5±0.5			NH
R. viscosa Vent.[2]	31.2±3.5	15.7±2.6	15.7±2.7	NH
**Galegeae**				
Colutea arborescens L.	10.9±2.3	10.9±2.3		NH
Glycyrrhiza echinata L.	8.8±1.6	8.8±1.6	9.9±1.4	NH
**Hedysareae**				
Caragana arborescens Lam.[2]	18.4±4.1	18.4±4.1		NH
Halimodendron halodendron (Pall.) Voss.[1]	12.2±2.8	12.2±2.8	16.3±1.8	NH
Onobrychis viciifolia Scop.	18.9±2.6	18.9±2.6		NH
**Cicereae**				
Cicer arietinum L.[4]	23.8±3.4	23.8±3.4	23.8±3.4	ANH
**Trifolieae**				
Trigonella foenum-graecum L.[4]	16.2±2.6			NH

T. gladiata M. Bieb.	11.1±2.9	13.7±2.0	13.7±2.0	NH
**Fabeae**				
Vicia faba L.[5]	35.7±1.1			ANH
V. cassubica L.	8.0±2.0			NH
V. cracca L.	6.9±1.8			NH
V. dumetorum L.	14.5±2.5			NH
V. grandiflora Scop.	9.8±2.4			NH
V. narbonensis L.	15.1±3.1			NH
V. pannonica Crantz	14.6±3.4			NH
V. pannonica Crantz ssp. *striata*	11.8±2.4	15.1±0.7		NH
V. pisiformis L.	9.6±2.0			NH
V. sativa L.[4]	10.4±1.8			NH
V. sativa L. ssp. *nigra* (L.) Ehrh.	15.4±3.0			NH
V. sepium L.	5.1±2.1			NH
V. sparsiflora Ten.	7.2±1.3			NH
V. tenuifolia Roth	1.8±0.4			NH
V. villosa Roth	8.3±2.1		14.4±0.4	NH
Lathyrus aphaca L.	11.4±2.3			NH
L. cicera L.[4]	18.1±3.3			NH
L. hirsutus L.	15.4±2.5			ANH
L. latifolius L.	7.7±2.1			ANH
L. niger (L.) Bernh.	19.2±3.4			NH
L. odoratus L.	10.7±2.8			ANH
L. pannonicus (Jacq.) Garcke	8.2±1.0	13.2±0.8		NH
L. pratensis L.	11.1±2.6			ANH
L. sativus L.	33.3±2.2			ANH
L. sylvestris L.	5.6±1.9			NH
L. sphaericus Retz.	13.1±2.9			NH
L. tuberosus L.	10.6±1.7			ANH
L. vernus (L.) Bernh.	19.5±3.8			NH
Lens culinaris Medik.[5]	18.7±1.7	18.7±1.7		ANH
Pisum sativum L.[5]	14.3±0.6	14.3±0.6		ANH
GRAMINEAE				
Sorgum bicolor (L.) Mönch	5.2±0.7	5.2±0.7		NH
Zea mays L.	22.0±1.0	22.0±1.0		NH
SAPINDACEAE				
Koelreuteria paniculata Laxm.	7.1±2.8	7.1±2.8		NH
TILIACEAE				
Tilia platyphyllos Scop.	4.4±1.5	4.4±1.5		NH

[1]Introduced, used as ornamentals; [2]Introduced and naturalised; [3]Cultivated occasionally as fodder; [4]Cultivated more-or-less regularly; [5]Cultivated regularly as fodder or human food; H = Host, ANH = acceptable non-host, NH = non-host. Species and authority names are given according to ILDIS (International Legume Database & Information Service) https://ildis.org/index.shtml [73]. Accessed: Jan. 2021

Additional file 1: Table S2 Developmental mortality of *A. obtectus* in seeds, and seed-coat thickness of naturally occurring leguminous non-host species with intact (I) or pre-drilled (PD) seed coats. No adults emerged from the seeds

Seed status	Plant family, tribe and species	Dead 1st instar without entering the seeds (%)[1]	Died within seeds as L1, L2, etc. of those entering (%)					Seed coat thickness (mm) (mean ±SE)
			L1	L2	L3	L4	Pupa	
	CAESALPINIACEAE							
	**Cercideae**							
I	*Cercis canadensis*	100	0	0	0	0	0	0.1
PD		17.4	82.6	0	0	0	0	±0.002
I	*C. siliquastrum*	100	0	0	0	0	0	0.09
PD		31.6	68.4	0	0	0	0	±0.002
	**Caesalpinieae**							
I	*Gleditsia delavayi*	90.9	9.1	0	0	0	0	0.19
PD		31.1	68.9	0	0	0	0	±0.009
I	*G. japonica*	100	0	0	0	0	0	0.28
PD		84.1	15.9	0	0	0	0	±0.01
I	*G. triacanthos*	100	0	0	0	0	0	0.23
PD		66.7	33.3	0	0	0	0	±0.007
	FABACEAE							
	**Genisteae**							
I	*Cytisophyllum sessilifolium*	100	0	0	0	0	0	0.17
PD		91.8	8.2	0	0	0	0	±0.01
I	*Cytisus scoparius*	100	0	0	0	0	0	0.15
PD		76.0	24.0	0	0	0	0	±0.01
I	*Laburnum alpinum*	100	0	0	0	0	0	0.15
PD		68.0	32.0	0	0	0	0	±0.009
I	*L. anagyroides*	100	0	0	0	0	0	0.15
PD		56.0	44.0	0	0	0	0	±0.006
I	*Lupinus multiflorus*	100	0	0	0	0	0	0.09
PD		64.4	35.6	0	0	0	0	±0.004
I	*L. polyphyllus*	100	0	0	0	0	0	0.09
PD		96.0	4.0	0	0	0	0	±0.005
I	*Petteria ramentacea*	100	0	0	0	0	0	0.16
PD		87.5	12.5	0	0	0	0	±0.008
I	*Spartium junceum*	100	0	0	0	0	0	0.16
PD		88.0	12.0	0	0	0	0	±0.009
	**Amorpheae**							
I	*Amorpha fruticosa*	100	0	0	0	0	0	0.13
PD		0	100					±0.01
	**Robinieae**							
I	*Robinia pseudoacacia*	100	0	0	0	0	0	0.16
PD		6.8	93.2	0	0	0	0	±0.006
I	*R. viscosa*	100	0	0	0	0	0	0.16
PD		4.0	96.0	0	0	0	0	±0.005
	**Galegeae**							
I	*Colutea arborescens*	92.0	8.0	0	0	0	0	0.20
PD		8.3	91.7	0	0	0	0	±0.01

I	*Glycyrrhiza echinata*	100	0	0	0	0	0	0.16
PD		72.0	28.0	0	0	0	0	±0.01
	**Hedysareae**							
I	*Caragana arborescens*	84.0	8.0	8.0	0	0	0	0.08
PD		8.7	91.3	0	0	0	0	±0.003
I	*Halimodendron halodendron*	100	0	0	0	0	0	0.16
PD		8.0	92.0	0	0	0	0	±0.006
I	*Onobrychis viciifolia*	88.0	12.0	0	0	0	0	0.09
PD		25.0	45.8	16.7	12.5	0	0	±0.005
	**Trifolieae**							
I	*Trigonella foenum-graecum*	100	0	0	0	0	0	0.13
PD		28.0	72.0	0	0	0	0	±0.01
I	*T. gladiata*	100	0	0	0	0	0	0.20
PD		58.3	41.7	0	0	0	0	±0.02
	**Fabeae**							
I	*Vicia cassubica*	100	0	0	0	0	0	0.11
PD		20.0	80.0	0	0	0	0	±0.004
I	*V. cracca*	100	0	0	0	0	0	0.12
PD		32.0	68.0	0	0	0	0	±0.009
I	*V. dumetorum*	100	0	0	0	0	0	0.11
PD		16	84	0	0	0	0	±0.002
I	*V. grandiflora*	100	0	0	0	0	0	0.06
PD		20.0	80.0	0	0	0	0	±0.003
I	*V. narbonensis*	100	0	0	0	0	0	0.19
PD		58.3	41.7	0	0	0	0	±0.007
I	*V. pannonica*	100	0	0	0	0	0	0.15
PD		44.0	56.0	0	0	0	0	±0.008
I	*V. pannonica* ssp. *striata*	100	0	0	0	0	0	0.12
PD		32.0	68.0	0	0	0	0	±0.002
I	*V. pisiformis*	100	0	0	0	0	0	0.16
PD		60.0	40.0	0	0	0	0	±0.006
I	*V. sativa*	98.9	1.1	0	0	0	0	0.08
PD		12.5	75.0	8.3	4.2	0	0	±0.005
I	*V. sativa ssp. nigra*	100	0	0	0	0	0	0.12
PD		60.0	40.0	0	0	0	0	±0.009
I	*V. sepium*	100	0	0	0	0	0	0.14
PD		9.5	90.5	0	0	0	0	±0.01
I	*V. sparsiflora*	100	0	0	0	0	0	0.13
PD		64.0	36.0	0	0	0	0	±0.006
I	*V. tenuifolia*	100	0	0	0	0	0	0.15
PD		20.0	80.0	0	0	0	0	±0.008
I	*V. villosa*	100	0	0	0	0	0	0.15
PD		56.0	44.0	0	0	0	0	±0.009
I	*Lathyrus aphaca*	100	0	0	0	0	0	0.12
PD		29.2	45.8	25.0	0	0	0	±0.01
I	*L. cicera*	100	0	0	0	0	0	0.09
PD		40.0	60.0	0	0	0	0	±0.002
I	*L. niger*	100	0	0	0	0	0	0.11
PD		0	96.0	4.0	0	0	0	±0.008
I	*L. pannonicus*	100	0	0	0	0	0	0.07
PD		16.0	76.0	4.0	4.0	0	0	±0.004
I	*L. sylvestris*	100	0	0	0	0	0	0.11
PD		12.0	84.0	4.0	0	0	0	±0.004
I	*L. sphaericus*	100	0	0	0	0	0	0.13

PD		8.0	92.0	0	0	0	0	±0.001
I	*L. vernus*	100	0	0	0	0	0	0.14
PD		20.0	80.0	0	0	0	0	±0.01
I	*Pisum sativum* (grown wild)	100	0	0	0	0	0	0.20
PD		40.0	44.0	16.0	0	0	0	±0.008

[1] Percentage of dead instars outside and inside seeds were calculated as follows: if all L1s were dead outside the seed, then 100% was entered in the 3rd column and 0% in the 4th. If, however, mortality outside seeds was <100%, then the L1s remaining alive and entering the seed together summed up to 100%, and this value was divided among further instars

Additional file 1: Table S3 Developmental mortality in seeds, adult emergence of *A. obtectus* from seeds, and seed coat thickness of plant selections of leguminous and non-leguminous species with intact (I) or pre-drilled (PD) seed coats

Seed status	Plant family, tribe, species/ cultivar and accession (numbered)	Dead 1st instar without entering the seeds (%)[1]	Died within seeds as L1, L2, etc. of those entering (%)					No. of adults emerged	Adult emergence (%)	Seed coat thickness (mm) (mean±SE)
			L1	L2	L3	L4	Pupa			
	FABACEAE									
	**Phaseoleae**									
I	*Glycine max*/Boly1[a]	100	0	0	0	0	0	0	0	0.10
PD		9.1	88.6	2.3	0	0	0	0	0	±0.005
I	*G. max*/Boly2 [a]	100	0	0	0	0	0	0	0	0.10
PD		27.3	72.7	0	0	0	0	0	0	±0.002
I	*G. max*/Boly3 [a]	87.8	12.2	0	0	0	0	0	0	0.08
PD		4.6	86.4	6.8	0	2.2	0	0	0	±0.005
I	*G. max*/Boly4 [a]	51.1	46.7	2.2	0	0	0	0	0	0.08
PD		4.5	95.5	0	0	0	0	0	0	±0.004
I	*G. max*/Boly5 [a]	97.7	2.3	0	0	0	0	0	0	0.08
PD		2.2	91.1	6.7	0	0	0	0	0	±0.004
I	*G. max*/Boly6 [a]	100	0	0	0	0	0	0	0	0.09
PD		2.2	97.8	0	0	0	0	0	0	±0.006
I	*G. max*/Boly7 [a]	100	0	0	0	0	0	0	0	0.09
PD		2.2	95.6	2.2	0	0	0	0	0	±0.003
I	*G. max*/Boly8 [a]	100	0	0	0	0	0	0	0	0.09
PD		2.2	97.8	0	0	0	0	0	0	±0.004
I	*G. max*/Boly9 [a]	95.6	2.2	0	0	0	0	1	2.2	0.09
PD		2.3	97.7	0	0	0	0	0	0	±0.005
I	*G. max*/Boly10 [a]	97.8	2.2	0	0	0	0	0	0	0.10
PD		0	100	0	0	0	0	0	0	±0.005
I	*G. max*/Boly12 [a]	95.2	4.8	0	0	0	0	0	0	0.10
PD		2.3	97.7	0	0	0	0	0	0	±0.004
I	*G. max*/ISz14 [a]	86.7	2.2	2.2	2.2	2.2	0	2	4.4	0.11
PD		2.3	18.2	40.9	18.2	15.9	0	2	4.5	±0.007
I	*G. max*/ISz15 [a]	93.2	0	0	0	2.3	0	2	4.5	0.10
PD		2.5	15.0	12.5	37.5	20.0	0	5	12.5	±0.003
I	*G. max*/ISz16 [a]	93.3	0	0	6.7	0	0	0	0	0.10
PD		0	23.7	15.8	36.8	10.5	0	5	13.2	±0.006
I	*G. max*/GSz3 [a]	100	0	0	0	0	0	0	0	0.08
PD		4.4	28.9	28.9	28.9	4.4	2.2	1	2.3	±0.004
I	*G. max*/Traverse[c]	52.6	18.4	5.3	10.5	5.3	0	3	7.9	0.10
PD		0	13.2	31.6	31.6	13.2	0	4	10.4	±0.005
I	*G. max*/Ewans[c]	100	0	0	0	0	0	0	0	0.09
PD		0	40.9	22.7	36.4	0	0	0	0	±0.005
I	*Vigna unguiculata*/ IFE Brown[c]	6.8	0	2.3	0	0	0	40	90.9	0.03
PD		0	0	2.3	0	0	0	43	97.7	±0.003
I	*V. unguiculata*/Boly [a]	58.1	2.3	0	0	0	0	17	39.6	0.05
PD		6.7	2.2	2.2	0	0	0	40	88.9	±0.002
I	*Phaseolus vulgaris*/ Budai piaci[c]	9.1	0	0	0	0	0	40	90.9	0.09
PD		20.0	0	0	0	0	0	36	80.0	±0.002

I	*P. vulgaris*/ Fehér szokvány[c]	62.2	0	0	0	0	0	17	37.8	0.07
PD		25.0	2.3	0	0	0	0	32	72.7	±0.002
I	*P. vulgaris*/Étkezési[c]	77.8	0	0	0	0	0	10	22.2	0.07
PD		35.6	0	0	0	0	0	29	64.4	±0.004
I	*P. vulgaris*/No.301[c]	70.5	2.3	0	0	0	0	12	27.2	0.09
PD		20.0	0	0	0	0	0	36	80.0	±0.004
I	*P. vulgaris*/ Kereskedelmi[c]	15.6	2.2	0	0	0	0	37	82.2	0.08
PD		2.4	0	0	0	0	0	41	97.6	±0.004
I	*P. vulgaris*/ Chevrier vert[c]	22.2	0	0	0	0	0	35	77.8	0.09
PD		2.4	0	0	0	0	0	40	97.6	±0.004
I	*P. vulgaris*/ Budai közép[c]	53.3	0	0	0	0	0	21	46.7	0.09
PD		6.7	0	0	0	2.2	0	41	91.1	±0.005
I	*P. vulgaris*/Harvester[c]	27.3	0	0	0	0	0	32	72.7	0.09
PD		4.5	0	0	0	0	0	42	95.5	±0.005
I	*P. vulgaris*/Amboy[c]	32.6	0	0	0	0	0	29	67.4	0.09
PD		13.9	0	0	0	0	0	37	86.1	±0.004
I	*P. vulgaris*/Pinto[c]	38.1	0	0	0	0	0	26	61.9	0.08
PD		6.7	4.4	0	0	0	0	40	88.9	±0.005
I	*P. vulgaris*/K1[c]	9.1	0	0	0	0	0	40	90.9	0.08
PD		4.4	0	0	0	0	2.2	42	93.4	±0.004
I	*P. vulgaris*/ Budai tarka[c]	0	4.6	0	0	0	0	41	95.4	0.09
PD		0	2.2	0	0	0	0	44	97.8	±0.005
I	*P. vulgaris*/Trobo[c]	13.6	0	0	0	0	0	38	86.4	0.1
PD		0	0	2.3	0	0	0	43	97.7	±0.004
I	*P. vulgaris*/ Tápiói cirmos[c]	13.6	0	0	0	0	0	38	86.4	0.09
PD		0	2.2	0	0	0	0	44	97.8	±0.005
I	*P. vulgaris*/Valja[c]	46.7	2.2	0	0	0	0	23	51.1	0.09
PD		9.3	0	0	0	0	0	39	90.7	±0.005
I	*P. vulgaris*/ Budai gömbölyű[c]	2.3	2.3	0	0	0	0	42	95.4	0.09
PD		0	4.5	0	0	0	0	42	95.5	±0.008
I	*P. vulgaris*/Constanca[c]	20.9	0	0	0	0	0	34	79.1	0.09
PD		2.3	2.3	0	0	0	2.3	40	93.1	±0.004
I	*P. vulgaris*/ Nagykállói[c]	27.9	4.7	0	0	0	0	29	67.4	0.09
PD		2.3	0	0	2.3	0	2.3	41	93.1	±0.004
I	*P. vulgaris*/F [a]	54.6	0	0	0	0	0	20	45.4	0.07
PD		0	0	0	0	0	2.2	44	97.8	±0.002
I	*P. vulgaris*/ Jabelyski stocnij[c]	7.1	2.4	0	0	0	0	38	90.5	0.09
PD		0	0	0	0	0	0	43	100	±0.007
I	*P. vulgaris*/Limetist[c]	71.4	0	0	0	0	0	12	28.6	0.1
PD		11.4	2.3	0	0	0	2.3	37	84.0	±0.002
	**Fabeae**									
I	*Vicia faba*/Gödöllői[c]	73.3	13.3	2.2	0	0	0	5	11.2	0.15
PD		53.5	4.6	0	0	0	0	18	41.9	±0.004
I	*V. faba*/K22 [a]	92.9	4.7	0	0	0	0	1	2.4	0.14
PD		36.4	25.0	2.3	0	0	4.5	14	31.8	±0.003
I	*V. faba*/K23 [a]	95.4	2.3	0	0	0	0	1	2.3	0.16
PD		51.3	10.3	0	0	2.6	0	14	35.8	±0.004

I	*V. faba*/K25 [a]	100	0	0	0	0	0	0	0	0.17
PD		20.9	30.2	2.3	0	9.3	0	16	37.3	±0.006
I	*V. faba*/K26 [a]	75.0	4.6	0	0	0	0	9	20.4	0.16
PD		30.3	4.6	4.6	0	0	0	26	60.5	±0.006
I	*V. faba*/K29 [a]	41.5	22.0	0	0	2.4	0	14	34.1	0.19
PD		34.9	18.6	2.3	0	2.3	2.3	17	39.5	±0.013
I	*Lens culinaris*/ Commercial[c]	100	0	0	0	0	0	0	0	0.05
PD		56.8	34.1	0	4.5	0	2.3	1	2.3	±0.001
I	*L. culinaris*/Értényi[c]	95.6	4.4	0	0	0	0	0	0	0.05
PD		34.9	55.9	0	2.3	2.3	2.3	1	2.3	±0.001
I	*Pisum sativum*/ Debreceni sötétzöld[c]	97.5	2.5	0	0	0	0	0	0	0.09
PD		11.6	0	20.9	23.3	7.0	2.3	15	34.9	±0.004
I	*P. sativum*/ Kelvedon csodája[c]	92.7	0	0	0	2.4	0	2	4.9	0.09
PD		25.6	2.3	7.0	9.3	0	2.3	23	53.5	±0.003
I	*P. sativum*/ Chrestensens gloriosa[c]	92.5	0	2.5	0	0	0	2	5.0	0.09
PD		31.8	0	34.1	9.1	2.3	2.3	9	20.4	±0.003
I	*P. sativum*/ Grüne Perle[c]	100	0	0	0	0	0	0	0	0.08
PD		32.6	0	2.3	4.6	0	0	26	60.5	±0.004
I	*P. sativum*/ Budai csemege[c]	100	0	0	0	0	0	0	0	0.08
PD		56.8	0	2.7	2.7	5.4	2.7	11	29.7	±0.002
I	*P. sativum*/ Rajnai törpe[c]	93.3	0	0	0	0	2.2	2	4.5	0.1
PD		11.4	2.3	2.3	0	2.3	2.3	35	79.4	±0.002
I	*P. sativum*/Maro[c]	55.6	0	0	2.2	4.4	0	17	37.8	0.09
PD		0	0	0	6.7	2.2	15.6	34	75.5	±0.004
I	*P. sativum*/ Újmajori kései[c]	93.3	0	0	2.2	0	0	2	4.5	0.08
PD		20.9	0	0	14.0	18.6	14.0	14	32.5	±0.003
I	*P. sativum*/Ruga[c]	86.8	0	2.2	4.4	4.4	2.2	0	0	0.07
PD		4.4	0	40.0	35.6	4.4	6.7	4	8.9	±0.003
I	*P. sativum*/Maxi[c]	83.7	0	2.3	2.3	4.6	0	3	7.1	0.07
PD		2.2	0	22.2	20.0	13.3	8.9	15	33.4	±0.003
I	*P. sativum*/Allround[c]	73.3	0	4.4	11.1	2.2	0	4	9.0	0.08
PD		2.2	2.2	46.7	26.7	6.7	0	7	15.5	±0.004
I	*P. sativum*/Birte[c]	77.3	0	13.6	2.3	0	0	3	6.8	0.07
PD		8.9	37.8	13.3	11.1	2.2	0	12	26.7	±0.002
I	*P. sativum*/Beli Uda[c]	97.8	0	0	0	0	2.2	0	0	0.07
PD		4.4	2.2	40.0	33.3	6.7	4.4	4	9.0	±0.002
I	*P. sativum*/Smaragd[c]	81.6	0	5.3	7.9	2.7	0	1	2.5	0.07
PD		18.9	0	2.7	43.2	16.2	2.7	6	16.3	±0.002
I	*P. sativum*/KZ30	79.5	0	0	5.1	2.6	0	5	12.8	0.08
PD		4.9	12.2	4.9	22.0	7.3	0	20	48.7	±0.004
I	*P. sativum*/KZ573	80.5	0	0	17.1	2.4	0	0	0	0.07
PD		7.3	7.3	22.0	31.7	17.1	2.4	5	12.2	±0.003
I	*P. sativum*/IP2 [a]	100	0	0	0	0	0	0	0	0.09
PD		4.9	9.8	2.4	2.4	7.3	2.4	28	71.8	±0.003
I	*P. sativum*/IP3 [a]	89.7	0	2.6	5.1	0	0	1	2.6	0.08
PD		11.1	0	31.1	11.1	8.9	4.4	15	33.4	±0.004
I	*P. sativum*/IP4 [a]	93.1	2.3	2.3	0	0	0	1	2.3	0.07
PD		13.3	0	33.3	17.8	2.2	4.4	13	29.0	±0.001

I	*P. sativum*/IP5 [a]	93.3	0	0	2.2	0	0	2	4.5	0.09
PD		14.3	0	47.6	16.7	7.1	0	6	14.3	±0.003
I	*P. sativum*/IP6 [a]	95.6	0	4.4	0	0	0	0	0	0.07
PD		6.7	0	75.6	15.6	0	0	1	2.1	±0.001
I	*P. sativum*/IP7 [a]	68.3	2.4	26.8	2.4	0	0	0	0	0.07
PD		7.1	2.4	66.7	16.7	2.4	0	2	4.7	±0.004
I	*P. sativum*/IP8 [a]	93.0	0	2.3	0	4.7	0	0	0	0.07
PD		4.6	4.6	47.7	25.0	6.8	2.3	4	9.0	±0.002
I	*P. sativum*/Bountiful[c]	93.2	0	2.3	4.6	0	0	0	0	0.1
PD		5.0	5.0	27.5	40.0	10.0	2.5	4	10.0	±0.002
I	*P. sativum*/Lincoln[c]	95.1	0	0	4.9	0	0	0	0	0.10
PD		37.6	7.5	45.0	10.0	0	0	0	0	±0.002
I	*P. sativum*/Iregi sárga[c]	95.5	0	4.5	0	0	0	0	0	0.10
PD		0	18.2	43.2	29.6	4.5	4.5	0	0	±0.002
I	*P. sativum*/ Gloire de Quimper[c]	100	0	0	0	0	0	0	0	0.08
PD		2.4	0	92.7	4.9	0	0	0	0	±0.003
	GRAMINEAE									
I	*Sorghum bicolor*/ Szegedi Törpe[c]	100	0	0	0	0	0	0	0	0.02
PD		42.2	57.8	0	0	0	0	0	0	±0.001
I	*Zea mays*/ Sze MSC 378 [a]	33.3	66.7	0	0	0	0	0	0	
PD		16.7	83.3	0	0	0	0	0	0	
	SAPINDACEAE									
I	*Koelreuteria paniculata*	100	0	0	0	0	0	0	0	0.39
PD		39.3	60.7	0	0	0	0	0	0	±0.01
	TILIACEAE									
I	*Tilia platyphyllos*	100	0	0	0	0	0	0	0	0.13
PD		13.3	86.7	0	0	0	0	0	0	±0.006

[1] Percentage of dead instars outside and inside seeds were calculated as follows: if all L1s were dead outside the seed, then 100% was entered in the 3rd column and 0% in the 4th. If, however, mortality outside was <100%, then the dead L1s outside plus all other developmental stages inside seeds were summed up to 100%, and divided among instars; [c]cultivar, [a]accession

Additional file 1: Table S4 Quartiles of adult emergence and mortality of stages of *A. obtectus* in seeds of host and acceptable non-host plant species, where more than one plant selection (cultivar and accession) or sample was evaluated. Medians are given in Table 4. *Glycine max* (17 plant selections), *Vigna unguiculata* (2), *Phaseolus vulgaris* (21), *Vicia faba* (6), *Lens culinaris* (2), *Pisum sativum* (27), and *Lathyrus tuberosus* 2 samples

Plant tribe and species[1]	Adult emergence (%) from seeds		L1 mortality (%)		Larval-to-pupal mortality (%)	
	Outside seeds		Inside seeds			
	Intact	Pre-drilled	Intact	Pre-drilled	Intact	Pre-drilled
**Phaseoleae**						
Glycine max		0-2.2	93.2-100	2.2-4.4	0-6.7	90.9-97.8
Vigna unguiculata	39.5-90.9	88.9-97.7	6.8-58.1	0-6.7	2.3-2.3	2.3-4.4
Phaseolus vulgaris	46.7-86.4	86.1-97.6	13.6-53.3	0-11.4	0-2.2	0-2.3
**Fabeae**						
Vicia faba	2.3-20.5	35.9-41.9	73.3-95.4	30.2-51.3	2.3-15.6	9.3-31.8
Lens culinaris		0-2.3	95.6-100	34.9-56.8	0-4.4	40.9-62.8
L. tuberosus		0-28.9		26.7-59.1		27.3-72.0
Pisum sativum	0-4.4	8.9-34.9	83.7-95.6	4.5-18.9	2.2-7.7	46.3-85.0

[1]According to ILDIS (International Legume Database & Information Service) https://ildis.org/index.shtml [73]. Accessed Jan. 2021

Additional file 1: Table S5 Nonparametric (Kendall's τ) correlation coefficients between seed traits and/or responses of *A. obtectus*

Correlations between seed traits and/or *A. obtectus* responses	H	ANH	NH
1. Seed mass *vs.* Number of eggs laid per female	0.0620	**0.0782**	**0.0580**
2. Seed coat thickness *vs.* L1 mortality outside seeds	**-0.3414**	**0.2139**	**0.2264**
3. Number of eggs laid per female *vs.* Adult emergence	0.1400	**0.3088**	-

H = hosts, ANH = acceptable non-hosts, NH = non-hosts. Bold numbers are significant correlations ($p<0.05$). Number of data in regressions: 1. H=154; ANH=399; NH=616; 2. H=22; ANH=55; NH=63; 3. H=44; ANH=110; NH=0

Additional file 1: Table S6 Chi^2-table and risk effect sizes with 95% confidence intervals (CIs) of *A. obtectus* L1 mortality due to seed coat thickness on hosts and acceptable non-host seeds (lumped together) *vs.* non-host seeds

Dependent variable	Independent variable: Seed coat thickness	<0.1 mm	>0.1 mm	Marg. sums	Effect size
L1 mortality outside seeds	L1 mortality <50%[1]	19	1	20	$p_{<50\%}$[3]= 0.95<br>$p_{>50\%}$[4]= 0.4917<br>RD[5] =0.4583; CI_{95}[6] = 0.30 & 0.62
	L1 mortality >50%[2]	59	61	120	RR[7] = 1.9322; CI_{95} = 1.57 & 2.38<br>OR[8] = 19.7; CI_{95} = 2.55 & 152.4<br>logit *d*[9] = 1.6441
	Marginal sums	78	62	140	Pearson correlation (φ) = 0.3229<br>Regression (φ^2) = 0.1043

[1]Hosts and acceptable non-hosts; [2]Non-hosts; [3]Proportion of L1 died in case on hosts and acceptable non-host seeds; [4]Proportion of L1 died in the presence of non-hosts; [5]Risk difference; [6]95% Confidence Interval; [7]Risk ratio; [8]Odds ratio; [9]Standardized mean difference; χ^2 for the table = 14.6, df =1, p<0.001

Additional file 1: Table S7 Chi^2-table and risk effect sizes of *A. obtectus* adult emergence from hosts and acceptable non-host seeds (lumped together) *vs.* non-host seeds

Dependent variable	Independent variable: Suitability (suit.) of cotyledon				Effect size
		More suit.	Less suit.	Marg. sums	
Adult emergence	>10%[1]	92	0	92	$p_{>10\%}$[3] = 0.9946<br>$p_{<10\%}$[4] = 0.2603<br>RD[5] =0.7343; CI_{95}[6] = 0.69 & 0.77
	<10%[2]	31	89	120	RR[7] = 3.8206; CI_{95} = 2.83 & 5.16<br>OR[8] = 523.4; CI_{95} = 348.7 & 785.6<br>logit *d*[9] = 3.4534
	Marginal sums	123	89	212	Pearson correlation (φ) = 0.7373<br>Regression (φ^2) = 0.5437

[1]Hosts and acceptable non-hosts; [2]Non-hosts; [3]Proportion of adults emerged from host seeds and acceptable non-host seeds; [4]Proportion of adults emerged from non-host seeds; [5]Risk difference; [6]95% Confidence Interval; [7]Risk ratio; [8]Odds ratio; [9]Standardized mean difference; χ^2 for the table = 117.61, df =1, p<0.001

Additional file 1: Table S8 Changes in the rank order of acceptable non-host (ANH) plant species in choice and in no-choice egg-laying tests, and ANH suitability order for larval *A. obtectus* development

Rank order in choice test[1]	Rank order in no-choice test[2]	Seed coat[3]	Rank order in larval development[4]
Phaseolus vulgaris	*Phaseolus vulgaris*		*Phaseolus vulgaris*
Vigna angularis	*Pisum sativum*		*Cicer arietinum*
Vicia faba	*Vigna angularis*		*Vigna unguiculata*
Vigna radiata	*Lupinus albus*		*Vigna radiata*
Glycine max	*Vigna radiata*		*Lathyrus sativus*
Lupinus albus	*Vicia sativa*		*Vigna angularis*
Pisum sativum	*Glycine max*		*Vicia faba*
Lathyrus sativus	*Vicia faba*		*Pisum sativum*
Lens culinaris	*Cicer arietinum*		*Lupinus albus*
Cicer arietinum	*Lens culinaris*		*Lens culinaris*
Vicia sativa	*Vigna unguiculata*		*Glycine max*
Vigna unguiculata	*Lathyrus sativus*		*Vicia sativa*

[1]Multiple choice tests were carried out as described in [29]. Three seeds of each of twelve ANH species and 100 females and 10 males were used/device in each of the 36 replicates of choice tests. [2]Egg-laying tests carried out as described in this paper. [3]The thin vertical line symbolizes the seed coat, which substantially modified acceptance and suitability. [4]Based on percentage of adult emergence. Mean number of eggs laid/female in the two types of test did not differ significantly (choice test: 22.3 ± 0.3, mean±SE, N=36, and no-choice test: 25.5 ± 1.6, N=93; *t*-test$_{1,\ 127}$=1.2653, p=0.2081)
